# Taking the RISC of exiting naïve pluripotency

**DOI:** 10.1186/s13059-016-0979-z

**Published:** 2016-05-16

**Authors:** Diana Guallar, Jianlong Wang

**Affiliations:** The Black Family Stem Cell Institute, Icahn School of Medicine at Mount Sinai, New York, NY 10029 USA; Department of Developmental and Regenerative Biology, Icahn School of Medicine at Mount Sinai, New York, NY 10029 USA; The Graduate School of Biomedical Sciences, Icahn School of Medicine at Mount Sinai, New York, NY 10029 USA

## Abstract

A new study shows how RNA-induced silencing complex (RISC)-mediated posttranscriptional regulation of chromatin remodelers allows for tight control of the naïve-to-primed pluripotency transition.

## Introduction

Pluripotent cells have the potential to give rise to any of the three germ layers of the embryo proper. In vivo, pluripotency is a transient state, but it can be perpetuated in vitro through derivation of embryonic stem cells (ESCs), from either the preimplantation inner cell mass (ICM) or the epiblast, and supplementation with exogenous signaling cues. Pluripotent cell lines can also be isolated from later developmental stages, such as the post-implantation epiblast. These epiblast-derived stem cells, known as EpiSCs, differ from ESCs in their culture conditions and in their more restricted differentiation potential. ESCs and EpiSCs represent two pluripotent states: naïve and primed, respectively.

Much has been learned about the transcriptional programs that play key roles in the maintenance of and exit from naïve pluripotency, but the posttranscriptional regulation of the transition out of naïve pluripotency remains largely unexplored. In their recent *Genome Biology* publication, Pandolfini and colleagues [1] describe their use of a global approach to characterize the transcriptional, posttranscriptional, and translational changes that occur during the first steps in the differentiation of mouse ESCs. In doing so, they establish a new paradigm in which the micro-RNA (miRNA)-mediated inhibition of the translation of a set of chromatin regulators plays a key role in the maintenance of ground-state pluripotency.

## Dynamic versus static experimental models for differentiation

**Fig. 1:**
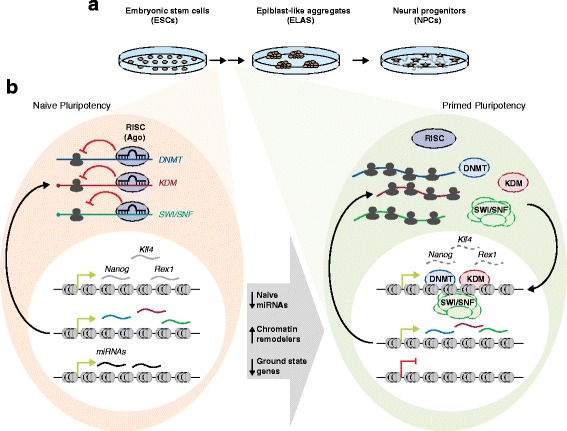
**a** In vitro differentiation model developed for this study. Embryonic Stem Cells (ESC) are induced to form Epiblast-Like Aggregates (ELA) which are similar to the post-implantation epiblast in vivo. ESC-to-ELA transition mimics early differentiation stages of cells from the inner cell mass of the blastocyst that give rise to epiblast cells after implantation. Further differentiation of ELA-to-neural progenitors can be achieved in vitro, and serve as a model to study later stages of differentiation. **b** RISC-mediated inhibition of translation of chromatin regulators during early priming. Naïve pluripotent stem cells (left chart) express Nanog, Klf4 and Rex1 genes. Chromatin remodelers such as DNA methyltransferases (DNMT), Lysine demethylases (KDM) and members of the SWItch/Sucrose Non-Fermentable complex (SWI/SNF) are expressed (mRNA depicted as wavy lines) both in naïve (left chart) and primed cells (right chart), but in naïve pluripotency they are translationally inhibited (ribosomes depicted in grey) through the RNA-induced silencing complex (RISC) and naïve specific miRNAs. Once cells become primed, naïve specific miRNAs are downregulated, allowing for the release from the RISC complex of the mRNAs coding for these chromatin regulators. Increased translation of DNMT, KDM and SWI/SNF proteins leads to the shutdown of ground pluripotency transcriptional programs, including genes such as Nanog, Klf4 and Rex1

Although in vitro ESC cultures are an invaluable research tool, it is clear that they may not behave in the same way as their in vivo counterparts [2]. Consequently, more dynamic systems of ESC differentiation that mimic the in vivo developmental process are preferred. Pandolfini and colleagues generated an early differentiation model in which ESCs were induced to differentiate into epiblast-like aggregates (ELA; Fig. 1) [1]. These cells, having just escaped ground-state pluripotency, had transcription profiles similar to those of post-implantation epiblast cells, as well as similar potential to differentiate and neuralize. The authors analyzed the transcriptional changes occurring in the ESC-to-ELA transition as a model for ICM-to-epiblast transition. They also examined variation in the load composition of the RNA-induced silencing complex (RISC), which uses miRNAs as templates for mRNA silencing, leading to reduced translation or to transcript degradation. Pandolfini and colleagues [1] showed for the first time that whereas global transcriptional changes mainly occur at later stages of ESC differentiation, specific translational regulation is characteristic of early differentiation priming. They identified new miRNA clusters, as well as the families of genes that are subjected to RISC-mediated control, that have important roles in naïve state maintenance and early differentiation. In the literature, there are ample examples of discordance between in vivo and in vitro phenotypes after ablation of a specific protein. Establishment of dynamic models that mimic in vivo development and studies of the underlying transcriptional and posttranscriptional regulatory mechanisms, such as the one highlighted here, will not only help to explain and reconcile such discrepancies but also provide molecular insights into early development.

## Chromatin remodeling during the pluripotency exit: the importance of timing

ESCs are characterized by an open chromatin structure and global hyper-transcription, with tight control of transcriptional ‘leakage’ [3, 4]. While ESCs can be maintained in the absence of chromatin repressors, primed EpiSCs are highly sensitive to the loss of these regulators [5], although the transcriptional level of these proteins is not greatly affected by the induction of differentiation. Interestingly, Pandolfini and colleagues [1] found that chromatin regulators and repressors are among the main targets of RISC-mediated translational inhibition in naïve ESCs [1]. In particular, they showed how the protein abundance of members of the DNA methyltransferase (DNMT), histone lysine demethylase (KDM), and SWItch/Sucrose Non-Fermentable nucleosome remodeling complex (SWI/SNF) families of epigenetic regulators are tightly regulated during the exit from ground-state pluripotency. Through the integration of RNAseq, polysome profiling, and the immunoprecipitation of Argonaute (Ago; the main RNA-binding RISC component), the authors elegantly show how RISC posttranscriptionally modulates the translation levels of these epigenetic regulators.

Once differentiation starts, RISC-loaded mRNAs of DNMT, KDM, and SWI/SNF proteins are coordinately released for translation; the resultant increase in their protein abundance shuts down the naive pluripotency transcriptional network, allowing cell differentiation (Fig. 1). Studies of the functional inhibition of DNMT, KDM, and SWI/SNF during the ESC-to-ELA transition showed that the activity of these chromatin regulators during priming is necessary for down-regulating both the naïve marker Nanog and markers of pluripotency (Klf4, Rex1, and Dax1) and for up-regulating priming markers [1]. Together, these results clearly show that the induction of chromatin modifiers is required for the naïve-to-primed transition.

## miRNAs and posttranscriptional regulation of pluripotency

Posttranscriptional regulation has been shown to play a key role in the maintenance of ground-state pluripotency. Two miRNA clusters, mmu-miR-290-295 and mmu-miR-302/365, are required for the maintenance of the naïve and primed states, respectively [6]. By analyzing RISC-loaded miRNAs and mRNAs, in combination with the quantification of cytoplasmic RNA abundance, Pandolfini and colleagues identified a new repertoire of miRNAs that are specific to the naïve and primed states, which will certainly be a great tool for future research [1]. The authors show that the translation of DNMT, KDM, and SWI/SNF is maintained at low levels or inhibited entirely in ESCs and that aberrant release from RISC-mediated miRNA repression causes destabilization of naïve pluripotency and upregulation of priming markers. This observation contrasts with the findings of previous studies of Dicer^-/-^ and Dgcr8^-/-^ ESCs, which have a hyper-naïve phenotype rather than a differentiated one in the absence of functional miRNA machinery [7]. Pandolfini and colleagues reconcile this discrepancy by showing that, in other studies, the in vitro depletion of Dicer does not account for the role of miRNAs in buffering gene expression in naïve pluripotent cells, nor does it allow the analysis of naturally occurring changes during cell priming. In contrast to previous studies of genetically ablated miRNA processors in established ESC lines [8, 9], the ESC-to-ELA transition model was able to recapitulate the requirement of Dicer function for pluripotency maintenance observed in vivo [7]. Further investigations will be needed to clarify how and when the RISC/miRNA machinery comes into play during the developmental priming process.

## Conclusions

Great efforts have been devoted to deciphering the signaling networks and epigenetic regulators that characterize naïve and primed pluripotent states in both mouse and human systems [10]. Most studies have focused on the transcriptional perturbations derived from the ablation of specific factors, but it is now clear that ESCs have an open-chromatin, widely transcribed genomic status that is maintained by mechanisms beyond transcriptional regulation. An increasing number of studies, including that of Pandolfini and colleagues, show that posttranscriptional regulation is as important as transcriptional regulation, or even more so, in preserving pluripotency. Developmental priming is associated with chromatin changes involving histone modifications and general remodeling of the nuclear architecture. The work by Pandolfini and colleagues represents important progress in explaining the interconnection between posttranscriptional control and chromatin remodeling regulation in differentiation (Fig. 1). The transient nature of pluripotent cells in the preimplantation embryo supports the existence of fast-responding control mechanisms that allow for both pluripotency exit and the establishment of defined somatic transcriptional programs. Only the integration of proteomic and transcriptomic data in new experimental models that mimic early differentiation will allow us to understand the complexity of embryonic development fully. This work by Pandolfini and colleagues is a reminder that it would be a risk not to take RISC into consideration while studying pluripotency and early development.

## Abbreviations

DNMT: DNA methyltransferase
ELA: epiblast-like aggregate
EpiSC: epiblast-derived stem cell
ESC: embryonic stem cell
ICM: inner cell mass
KDM: histone lysine demethylase
miRNA: micro-RNA
RISC: RNA-induced silencing complex
SWI/SNF: SWItch/Sucrose Non-Fermentable nucleosome remodeling complex
